# Reduction of *Clostridium Difficile *and vancomycin-resistant *Enterococcus *contamination of environmental surfaces after an intervention to improve cleaning methods

**DOI:** 10.1186/1471-2334-7-61

**Published:** 2007-06-21

**Authors:** Brittany C Eckstein, Daniel A Adams, Elizabeth C Eckstein, Agam Rao, Ajay K Sethi, Gopala K Yadavalli, Curtis J Donskey

**Affiliations:** 1 Research Service, Louis Stokes Cleveland Veterans Affairs Medical Center, 10701 East Blvd., Cleveland, Ohio, USA; 2 Infection Control Department, Louis Stokes Cleveland Veterans Affairs Medical Center, 10701 East Blvd., Cleveland, Ohio, USA; 3 Department of Medicine, University Hospitals of Cleveland, 10000 Euclid Avenue, Cleveland, Ohio, USA; 4 Department of Epidemiology and Biostatistics, Case Western Reserve University School of Medicine, 10900 Euclid Avenue, Cleveland, Ohio, USA

## Abstract

**Background:**

Contaminated environmental surfaces may play an important role in transmission of some healthcare-associated pathogens. In this study, we assessed the adequacy of cleaning practices in rooms of patients with *Clostridium difficile*-associated diarrhea (CDAD) and vancomycin-resistant *Enterococcus *(VRE) colonization or infection and examined whether an intervention would result in improved decontamination of surfaces.

**Methods:**

During a 6-week period, we cultured commonly touched surfaces (i.e. bedrails, telephones, call buttons, door knobs, toilet seats, and bedside tables) in rooms of patients with CDAD and VRE colonization or infection before and after housekeeping cleaning, and again after disinfection with 10% bleach performed by the research staff. After the housekeeping staff received education and feedback, additional cultures were collected before and after housekeeping cleaning during a 10-week follow-up period.

**Results:**

Of the 17 rooms of patients with VRE colonization or infection, 16 (94%) had one or more positive environmental cultures before cleaning versus 12 (71%) after housekeeping cleaning (p = 0.125), whereas none had positive cultures after bleach disinfection by the research staff (p < 0.001). Of the 9 rooms of patients with CDAD, 100% had positive cultures prior to cleaning versus 7 (78%) after housekeeping cleaning (p = 0.50), whereas only 1 (11%) had positive cultures after bleach disinfection by research staff (p = 0.031). After an educational intervention, rates of environmental contamination after housekeeping cleaning were significantly reduced.

**Conclusion:**

Our findings provide additional evidence that simple educational interventions directed at housekeeping staff can result in improved decontamination of environmental surfaces. Such interventions should include efforts to monitor cleaning and disinfection practices and provide feedback to the housekeeping staff.

## Background

Patients colonized or infected with healthcare-associated pathogens often shed these organisms onto their skin and into the environment [1]. Although direct contact with patients is generally considered the major source for acquisition of pathogens on healthcare workers' hands and subsequent transmission to other patients, several recent studies suggest that contaminated environmental surfaces may also play an important role in pathogen transmission [2-10]. For example, we found that vancomycin-resistant enterococci (VRE) and *Staphylococcus aureus *were frequently acquired on hands of investigators after contact with contaminated objects such as bed rails and bedside tables in colonized patients' rooms [2,3]; daily disinfection of environmental surfaces in VRE-colonized patients' rooms was associated with reduced acquisition of VRE on investigators' hands [2]. In a medical intensive care unit, Hayden et al [4] found that enforcing routine environmental cleaning measures was associated with decreased VRE contamination on surfaces and healthcare workers' hands, and also with a significant reduction in VRE cross-transmission. In two other recent reports, control of VRE outbreaks has been attributed in part to implementation of a program of environmental decontamination [5,6]. Environmental decontamination with 10% bleach has also been associated with reductions in *Clostridium difficile *infections [9,10].

Beginning in 2002, our institution has experienced a significant outbreak of *C. difficile*-associated disease (CDAD) that has been associated with emergence of the recently described epidemic strains in the Cleveland area [[11,12], and authors' unpublished data]. Despite control measures including contact precautions for patients with documented and confirmed CDAD and environmental disinfection of CDAD patients' rooms with 10% bleach, we continued to experience high rates of infection (~15 CDAD cases/1,000 patient discharges). Because we and others have demonstrated that frequently touched objects in patients' rooms may not be adequately disinfected by housekeeping staff [3,13], we assessed the adequacy of cleaning practices for CDAD rooms in our institution and examined whether an intervention would result in improved decontamination of frequently touched surfaces. For comparison, we also examined the adequacy of cleaning practices in rooms of patients colonized or infected with VRE because these rooms receive routine terminal cleaning after patient discharge.

## Methods

### Setting

The Cleveland Veterans Affairs Medical Center is a 368-bed acute care medical facility. Patients with CDAD are placed in contact precautions until diarrhea has resolved. Two years prior to the start of the study, the infection control department instituted bleach disinfection as a control measure for *C. difficile*. The housekeeping staff was in-serviced on the *C. difficile *outbreak and the importance of their role in preventing transmission was emphasized. The housekeepers were instructed to use 10% bleach solution (Sunstorm, State Chemical, Cleveland, OH) for terminal disinfection of CDAD patients' rooms. Terminal disinfection was to be performed using a clean cloth or mop soaked in 10% bleach, and it was stressed that frequently touched objects such as bed rails, bedside tables, and call buttons were to be disinfected. Subsequently, the housekeeping staff was periodically contacted to reinforce the bleach disinfection policy.

Patients with VRE colonization or infection were included as a comparison group because they are currently managed with standard precautions and routine terminal cleaning is performed. According to the housekeeping staff, routine terminal disinfection was to include bed rails and bedside tables, but telephones, call buttons, and door handles were not cleaned unless they were obviously soiled. The disinfectant used for routine decontamination of patient rooms is Super HDQ Neutral (Spartan Chemical Company, Inc., Maumee, OH), which is a quaternary ammonium compound.

### Pre-intervention assessment of cleaning practices

A prospective 6-week before-after study was performed to assess the adequacy of terminal cleaning and disinfection practices in rooms of patients with CDAD and VRE colonization or infection. All patients with known CDAD and VRE colonization or infection during the study period were considered for enrollment; however, only those patients who were discharged between 9 AM and 5 PM from Monday to Friday were included in the assessment of housekeeping cleaning practices. Standardized chart review was performed to collect information regarding demographics, medical conditions, and culture results. The hospital's Institutional Review Board approved the study protocol.

Baseline cultures of environmental surfaces were obtained within 3 days of patient discharge from the room. Six sites were cultured in each room, including the bedrail, telephone, call button, door knob, toilet seat, and bedside table. Two sterile, pre-moistened cotton-tipped swabs were applied directly onto the selected surfaces in a uniform fashion. For rooms of VRE-colonized patients, one swab was plated directly onto Enterococcosel agar (Becton Dickinson and Company, Sparks, MD) containing 20 μg/mL of vancomycin and the other swab was placed directly into Enterococcosel broth (Becton Dickinson) containing 20 μg/mL of vancomycin. The plates and broth enrichment cultures were incubated for 48 hours at 37°C; broth cultures were then plated onto Enterococcosel agar containing 20 μg/mL of vancomycin and incubated another 48 hours. Colonies with unique morphology were subjected to identification and susceptibility testing in accordance with Clinical Laboratory Standards Institute guidelines [14].

For rooms of CDAD patients, the swabs were placed into an eppendorf tube and transferred to an anaerobic chamber within 1 hour of collection (Coy Laboratories, Grass Lake, MN). One swab was directly plated onto cycloserine-cefoxitin-fructose agar containing 0.1% taurocholic acid (CCFA-TA) and the other was placed into 300 μl of CCF broth containing 0.1% taurocholic acid and incubated for 48 hours prior to plating onto CCFA-TA. Plates were incubated for 72 hours at 37°C. Isolates were confirmed to be *C. difficile *on the basis of typical odor and appearance of colonies and by a positive reaction using Pro Disk (Key Scientific Products, Round Rock, TX). *C. difficile *isolates were tested for in-vitro cytoxin production using *C. difficile *Tox A/B II (Wampole Laboratories, Princeton, NJ), and isolates that did not produce toxin were not included in the number of positive cultures.

In order to assess the adequacy of housekeeping cleaning and disinfection practices, cultures of the same surfaces in the rooms were obtained after terminal cleaning was performed by the housekeeping staff, but prior to admission of another patient. Finally, in order to confirm the efficacy of 10% bleach for decontamination of *C. difficile *and VRE, one of us (B.C.E.) disinfected the same surfaces using a 10% bleach solution (Dispatch, Caltech Industries, Inc. Midland, MI). The surfaces were wiped with a cloth soaked with 10% bleach to provide physical removal of dirt or other substances and sprayed with bleach to ensure that the surfaces were thoroughly wet. The surfaces were allowed to air dry. Cultures were then obtained from the same surfaces.

### Intervention and post-intervention assessment

After completion of the initial assessment of adequacy of housekeeping cleaning, the research team presented their findings to the housekeeping staff. Education was provided regarding the importance of environmental cleaning as a means to reduce transmission of pathogens. The importance of cleaning and disinfecting frequently touched surfaces was emphasized. In addition, the housekeeping staff was encouraged to provide input regarding measures that might improve their ability to perform adequate terminal cleaning. To assess the effect of the intervention, additional cultures were obtained before and after the housekeeping staff performed terminal cleaning of rooms of several patients with CDAD or VRE colonization or infection during the 10-week period after the intervention.

### Statistical analysis

Data were analyzed using STATA 9.0 (StataCorp, College Station, TX). In describing the study population, continuous data were analyzed using unpaired *t *tests and categorical data were assessed using Fisher's exact test. In evaluating the effectiveness of the intervention, an exact McNemar's chi-square test was used to compare VRE and *C. difficile *culture positivity in rooms and on specific surfaces prior to and after cleaning by housekeeping staff. This test of significance was used to then compare culture positivity after cleaning by housekeeping staff and after cleaning by research staff.

## Results

### Pre-intervention assessment of cleaning practices

Prior to the intervention, we studied 17 patients with VRE colonization and 9 patients with CDAD. All of the patients were male. The average ages of the VRE and CDAD patients were 70.0 (range 42 to 86) and 63.8 (range 28 to 80), respectively. Of the 17 patients with VRE colonization, 5 (29%) were incontinent of feces, 6 (35%) had diarrhea, 6 (35%) were receiving antibiotic therapy, 11 (64%) were in multiple-bed rooms, and 4 (24%) were in an intensive care unit. Of the 9 patients with CDAD, all were in private rooms and were receiving therapy for *C. difficile*, 4 (44%) were incontinent of feces, and 1 (11%) was in an intensive care unit.

**Figure 1:**
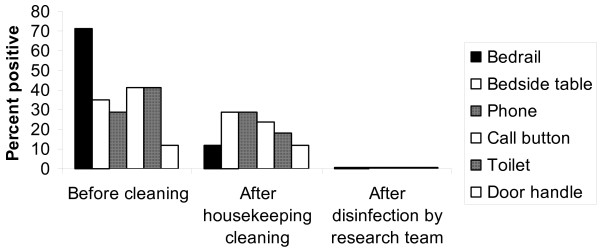
Percentage of positive environmental cultures for vancomycin-resistant *Enterococcus *(VRE) before and after housekeeping cleaning and after disinfection with 10% bleach by the research team. Seventeen rooms of patients with VRE colonization or infection were cultured.

**Figure 2:**
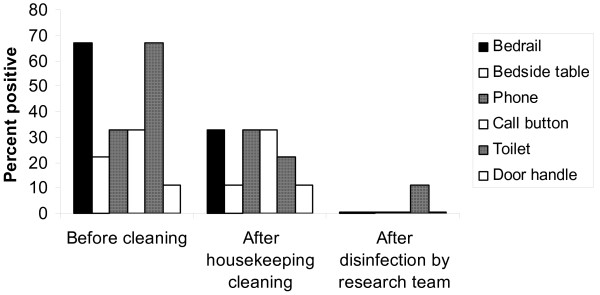
Percentage of positive environmental cultures for *Clostridium difficile *before and after housekeeping cleaning and after disinfection with 10% bleach by the research team. Nine rooms of patients with *Clostridium difficile*-associated disease were cultured.

Figures 1 and 2 show the percentage of positive broth-enrichment environmental cultures before and after housekeeping cleaning and after disinfection with 10% bleach by the research team for VRE and *C. difficile*, respectively. Of the 17 rooms of patients with VRE colonization or infection on at least one of the surfaces surveyed, 16 (94%) had one or more positive environmental cultures before cleaning versus 12 (71%) after housekeeping cleaning (p = 0.125), whereas none had positive cultures after bleach disinfection by the research staff (p < 0.001). Overall, 72 of 102 total environmental cultures (71%) were positive for VRE before cleaning and 58 of 102 (57%) were positive after housekeeping cleaning. Housekeeping staff were effective in disinfecting bedrails (p =0.002) but not substantially so for other surfaces. Of the 9 rooms of patients with CDAD, 100% had one or more positive cultures prior to cleaning versus 7 (78%) after housekeeping cleaning (p = 0.50), whereas only 1 (11%) had a single positive culture from a toilet after research bleach disinfection (p =0.031). Overall, 30 of 54 (56%) total environmental cultures were positive for *C. difficile *before cleaning and 24 of 54 (44%) were positive after housekeeping cleaning.

**Figure 3:**
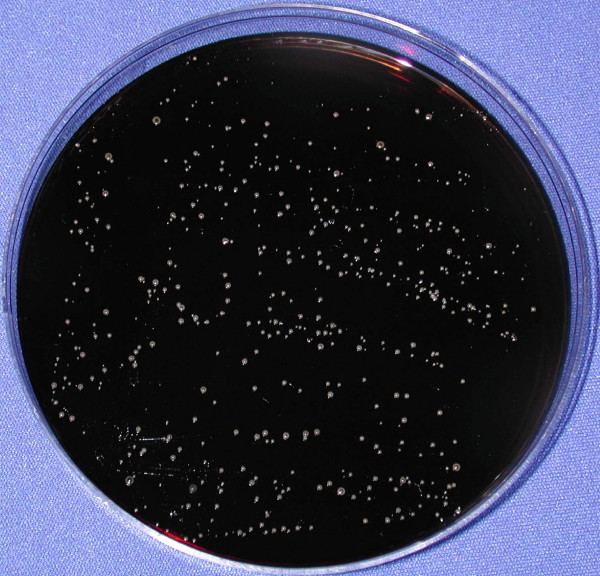
Culture plate showing gross contamination of a call button with vancomycin-resistant *Enterococcus *(VRE) after completion of cleaning by housekeeping staff. The patient was a 68 year-old man with *Clostridium difficile*-associated disease and VRE intestinal colonization. A sterile, pre-moistened cotton-tipped swab was applied to the surface of the call button and directly plated onto Enterococcosel agar containing 20 μg per mL of vancomycin. The same call button yielded *C. difficile *by broth enrichment culture.

For both *C. difficile *and VRE, broth enrichment cultures yielded about twice as many positive cultures than the direct plating method. All cultures that were positive by direct plating were also positive by broth-enrichment. For *C. difficile*, the number of colonies recovered by direct plating onto CCFA-TA plates was low (median, 3; range, 1 to 25 colonies). In contrast, many of the VRE cultures that were positive by direct plating yielded high levels of organisms that were too numerous to count; Figure 3 shows an illustration of gross contamination of a call button with VRE after completion of cleaning by housekeeping staff.

### Intervention and post-intervention assessment

As noted previously, the intervention was initiated with a presentation of the initial culture findings at a housekeeping staff meeting followed by discussion of factors that impacted their ability to perform thorough cleaning. The housekeeping staff expressed concern that the time permitted for terminal cleaning was not adequate and that there were often significant delays before they were notified that a room was ready to be cleaned. The hospital administration agreed to allow the housekeeping staff additional time for terminal cleaning (30 minutes/room) and the nursing staff was asked to provide more rapid notification of when rooms were ready for cleaning. The housekeeping staff also expressed concern that the cleaning agents being used might not adequately kill the pathogens. We therefore performed in vitro studies on a laboratory bench top to assess killing of *C. difficile *spores and VRE by the cleaning agents used in our hospital. Freshly-prepared Sunstorm bleach, Sunstorm obtained from housekeeping carts, and Dispatch bleach efficiently killed an inoculum of ~10^6 ^colony-forming units of *C. difficile *spores or VRE in 10 μl of phosphate-buffered saline that was allowed to air dry on the surfaces; the contaminated surfaces were disinfected and cultured in the same manner as described for the environmental surfaces in rooms. Super HDQ disinfectant killed VRE but not *C. difficile *spores (data not shown).

After the intervention, the housekeeping staff agreed to apply 10% bleach solution to disinfect frequently touched surfaces (e.g. bed rails, bedside tables, call buttons, telephones, toilet seats, door handles) in rooms of CDAD patients using a spray bottle. The surfaces were then allowed to air-dry. In addition, without prompting from the research team, the housekeeping staff elected to incorporate this method of bleach disinfection of commonly touched objects into their terminal cleaning practices for all patient rooms in our facility. Although there were initially concerns that bleach might cause damage to surfaces in the rooms, no complaints regarding such damage have been reported. In addition, interviews with the housekeeping staff were conducted, and no complaints related to the application of bleach were reported.

To assess the effect of the intervention, we performed environmental cultures before and after housekeeping cleaning in rooms of 10 patients with VRE colonization or infection and 10 patients with CDAD during the 4-month period after the intervention. Eight (80%) rooms of patients with VRE colonization or infection had one or more positive environmental cultures before cleaning versus 0 (0%) after housekeeping cleaning (p < 0.001). Nine (90%) of rooms of patients with CDAD had one or more positive cultures prior to cleaning versus 2 (20%) after housekeeping cleaning (p < 0.01). The Infection Control Department currently meets monthly with the housekeeping staff to provide feedback regarding culture results and to maintain awareness of the importance of environmental cleaning as a means to control healthcare-associated pathogens.

## Discussion

Two years prior to our study, the infection control department instituted a bleach disinfection program as a control measure for *C. difficile*. The housekeeping staff was in-serviced, and it was stressed that frequently touched objects such as bed rails, bedside tables, and call buttons were to be disinfected. Despite the initial intervention and periodic reminders of the policy, we found that frequently touched surfaces in rooms of patients with CDAD were often contaminated after terminal cleaning and disinfection by housekeeping staff. Similar results were obtained in rooms of patients with VRE colonization or infection. An educational intervention was well-received by the housekeeping staff and resulted in modification of cleaning practices and improved decontamination of frequently touched surfaces. Our findings provide additional evidence that simple interventions can result in improved environmental decontamination in healthcare institutions.

Our experience also demonstrates that environmental cleaning interventions should include some means of monitoring the efficacy of decontamination with feedback to the housekeeping staff. Such monitoring of cleaning performance has been recommended in recently published guidelines from the Centers for Disease Control and Prevention and the Society for Healthcare Epidemiology of America [15-17]. Although our intervention included environmental cultures, less labor-intensive methods of monitoring cleaning practices may also be effective. For example, Carling et al [13] used an invisible fluorescent marker to monitor cleaning practices; feedback to housekeeping staff led to a sustained improvement in cleaning of surfaces. Hayden et al [4] performed environmental cultures, but also observed cleaning practices and provided feedback to housekeepers.

Our study has several limitations. First, only one hospital was included, and our findings may not be applicable to all institutions. Second, we monitored the effect of the intervention on cleaning practices for only 4 months for the purposes of the study. However, we intend to continue ongoing monitoring of environmental disinfection practices because we believe that such monitoring will be essential to maintain compliance. Third, molecular typing was not performed to confirm that the organisms recovered from surfaces were genetically related to isolates recovered from the stool of patients occupying the rooms. Fourth, although bleach is required for disinfection of CDAD patients' rooms, it should be noted that non-sporicidal disinfectants are equally effective for elimination of other pathogens (i.e., the improvement in disinfection of VRE by housekeeping is likely attributable to improved performance and would have been achieved with agents other than bleach). Fifth, our disinfection method was performed after patients were discharged from the rooms and included spraying of bleach solution onto surfaces; wiping with a soaked cloth is preferred if patients or healthcare workers are present to limit exposure to bleach. Finally, we have not included an assessment of the impact of the intervention on rates of CDAD. Such an assessment will be performed after monitoring rates of CDAD over a longer period of time.

## Conclusion

Frequently touched surfaces in rooms of patients with CDAD or VRE colonization or infection were often contaminated with these pathogens after terminal cleaning by housekeeping staff. Simple educational interventions directed at housekeepers can result in improved environmental decontamination, but these interventions should include efforts to monitor cleaning practices and provide feedback to the housekeeping staff.

## Competing interests

The author(s) declare that they have no competing interests.

## Authors' contributions

CJD conceived of the study, participated in drafting the manuscript, and edited the manuscript. BCE performed the cultures and data collection and assisted in drafting the manuscript. DAA contributed to the study design and assisted with cultures and data collection. ECE contributed to study design and assisted with the intervention. AR assisted with performance of cultures and data collection. GKY contributed to study design and assisted in editing the manuscript. AKS performed the statistical analyses and assisted in editing the manuscript. All authors read and approved the final manuscript.

## Pre-publication history

The pre-publication history for this paper can be accessed here:


